# The impact of acetylsalicylic acid dosed at bedtime on circadian rhythms of blood pressure in the high-risk group of cardiovascular patients—a randomized, controlled trial

**DOI:** 10.1007/s00228-020-02997-8

**Published:** 2020-09-21

**Authors:** Beata Krasińska, Lech Paluszkiewicz, Ewa Miciak-Ławicka, Maciej Krasinski, Piotr Rzymski, Andrzej Tykarski, Zbigniew Krasiński

**Affiliations:** 1 Department of Hypertension, Angiology and Internal Diseases, Poznan University of Medical Sciences, Długa 1/2, 61-848 Poznań, Poland; 2 Department of Thoracic And Cardiovascular Surgery/Perioperative diagnostics Bad Oeynhausen, Heart and Diabetes Center NRW, Ruhr-University of Bochum, Bochum, Germany; 3 Imperial College London School of Medicine, London, UK; 4 Department of Environmental Medicine, Poznan University of Medical Sciences, Poznan, Poland; 5 Integrated Science Association (ISA), Universal Scientific Education and Research Network (USERN), Poznan, Poland; 6 Department of General and Vascular Surgery, Poznan University of Medical Sciences, Poznan, Poland

**Keywords:** Acetylsalicylic acid, Anti-hypertensive effect, Bedtime administration, Chronotherapy, Circadian rhythm, Randomized controlled trial

## Abstract

**Purpose:**

Time of drug administration may significantly influence its effect. The aim of the present study was to investigate the effect of ASA (administrated in the morning or in the evening) on the anti-hypertensive effect and diurnal blood pressure profile in the high-risk group of cardiovascular patients.

**Methods:**

All patients (*n* = 114) had been diagnosed with coronary heart disease and arterial hypertension prior to the enrolment and had been treated with 75 mg per day of ASA in the morning. The patients were randomly assigned to one of the two study groups receiving 75 mg of ASA per day in a single antiplatelet therapy for 3 months in the morning (*n* = 58) or in the evening (*n* = 56). The control group (*n* = 61) consisted of patients with arterial hypertension but without coronary heart disease, not receiving ASA. In all the patients, during each visit, clinical blood pressure (BP) and ambulatory blood pressure measurements (ABPM) were performed.

**Results:**

There was a significant reduction in 24-h BP and blood pressure at night in the ASA group evening group compared with the ASA morning group and the control group.

**Conclusions:**

The present study demonstrated that compared with the use of ASA in the morning, its administration in the evening may lead to favourable drop in the ABPM and an improvement of the diurnal profile in the high-risk group of cardiovascular patients who are not naïve to ASA.

**Electronic supplementary material:**

The online version of this article (10.1007/s00228-020-02997-8) contains supplementary material, which is available to authorized users.

## Introduction

Acetylsalicylic acid (ASA) is one of the most commonly used drugs in the secondary prevention of coronary arterial disease [1–3]. Nowadays a lot of attention is drawn to the its effect on vascular endothelium in which it inhibits the expression of pro-inflammatory cytokines and leukocyte adhesives, increases the production of nitric oxide, and consequently leads to vasodilatation [4–6]. This effect is further increased by the reduction of production of vasoconstrictive compounds, such as endothelin, thromboxane A2, and prostaglandin. Both of the above-described mechanisms may have an effect on lowering blood pressure, which suggests a pleiotropic mechanism of action of ASA independent of the effect on cyclooxygenase type 1 [4–6]. Patients showing a proper blood pressure drop at night (by 10–20%) are referred to as dippers [7–10]. A small blood pressure drop characterizes the non-dippers profile at night (< 10%). This group of patients has an increased incidence of organ damage and an increased risk of cardiovascular complications of arterial hypertension, such as left ventricular hypertrophy and ventricular arrhythmias [11, 12]. Increased systemic pressure at night causes carotid remodelling and glomerular damage, which result in albuminuria and the development of renal failure [13–16]. However low-dose, ASA administration has been documented to exert preventive effects on major cardiovascular events in hypertensive subjects, some studies found no effect on blood pressure profile [17, 18]. One should note that in most cases, ASA was administrated as a single dose in the morning hours or time of its use was not reported [4, 18–20]. It is now well established that time of drug administration may profoundly influence the effects it is aimed to exert, and this phenomenon also includes anti-hypertensive drugs [7, 21, 22]. This is due to circadian rhythms, which are governed by a network of hierarchical master clocks present at various locations in the brain and peripheral tissues [23]. Such circadian variation has been also demonstrated for factors such as serum nitric oxide, prostaglandin, plasma renin activity, angiotensin II, and angiotensin-converting enzyme [24–26]. Taking this into consideration it could be hypothesised whether administration of ASA in the morning or evening may differentially affect the change in the diurnal blood pressure profile. To date, only a few studies have addressed the relationship between the time of taking ASA and blood pressure. It was however demonstrated that anti-platelet effect of ASA is more significant after administration in the evening. Moreover, a recent large, multicentre, controlled, prospective endpoint trial has clearly showed that a routine administration of hypotensive medications at bedtime, as opposed to upon waking, results in improved control of blood pressure in hypertensive patients [27]. Nevertheless, in most cases, ASA is taken in one daily dose in the morning hours [28, 29].

Therefore, the aim of the present study was to investigate the effect of ASA (administrated in the morning or in the evening) on the anti-hypertensive effect and diurnal blood pressure profile in the high-risk group of cardiovascular patients with coronary heart disease and arterial hypertension.

## Material and methods

The study included 175 patients (59 women and 116 men), aged 59.8 years who were admitted to the Department of Hypertension. The recruitment period was 21 months (from May 2015 to January 2017). From the recruited group, 114 patients had been diagnosed with coronary heart disease (confirmed by angiographic examination) and arterial hypertension prior to the enrolment and had been treated with 75 mg per day of enteric-coated ASA (Acard, Polfa, Poland) in the morning. These patients (*n* = 114) were randomly assigned, in a blinded fashion (a sealed opaque envelope principle), to one of the two study groups receiving 75 mg of ASA per day in a single antiplatelet therapy in the morning (*n* = 58) or in the evening (*n* = 56). To ensure compliance with taking ASA at the specific time, the patients were instructed by the researchers and each patient received a written schedule of drug administration. The control group included 61 patients with arterial hypertension, without coronary heart disease, and not receiving any antiplatelet drugs (Table 1). The study was approved by the Bioethics Committee of Poznan University of Medical Sciences (Approval No. 373/15). Each participant signed informed consent to participate in the study. Exclusion criteria for the study were as follows: secondary hypertension, myocardial infarction and stroke within 6 months prior to the study, chronic heart failure NYHA III and IV, chronic kidney disease (glomerular filtration rate < 60 mL/min), addiction to alcohol and psychotropic substances, active cancer. Additional exclusion criteria for the study group were: confirmed hypersensitivity to ASA, history of bleeding due to ASA, taking clopidogrel or other antiplatelet agents, haemorrhagic diathesis, active gastric and/or duodenal ulcer disease, hypersensitivity to the active substance: ASA, other salicylates, or any component of the drug, using non-steroidal anti-inflammatory drugs. The exclusion criterion for the control group was the use of ASA preparations in the last 30 days. There were no changes in the concomitant treatment (lipid lowering, anti-hypertensive, and antidiabetic), and no non-steroidal anti-inflammatory drugs were taken during the study.

**Table 1:** Demographic characteristics of the patients. Different superscript letter (a, b) denote significant differences between groups in each row (*p* < 0.05 in multiple comparison post-hoc Dunn test following Kruskal-Wallis ANOVA). Superscript † denotes statistically significant difference (*p* = 0.0000002) in Aspirin Reaction Units between Visit 1 and Visit for ASA evening group

	ASA morning (*n* = 58)	ASA evening (*n* = 56)	Control group (*n* = 61)	*p* value	Statistics
Female/male (*n*)	19/39	19/37	21/40	> 0.05	Chi-square
Smokers/non-smokers (*n*)	14/44	17/39	17/44	> 0.05	
Age (years) (mean ± SD)	59.8 ± 7.6	60.3 ± 7.1	59.9 ± 7.1	> 0.05	Kruskal-Wallis ANOVA
Weight (kg) (mean ± SD)	84.4 ± 9.7	84.9 ± 12.9	84.1 ± 10.1	> 0.05	
Height (m) (mean ± SD)	1.69 ± 0.1	1.69 ± 0.1	1.69 ± 0.1	> 0.05	
Body mass index (kg/ m2) (mean ± SD)	29.52 ± 3.95	29.50 ± 4.4	29.52 ± 4.1	> 0.05	
Waist (cm) (mean ± SD)	93.1 ± 11.1	92.5 ± 11.4	93.7 ± 11.6	> 0.05	
Hip (cm) (mean ± SD)	105.2 ± 10.2	105.3 ± 12.3	105.2 ± 10.6	> 0.05	
Waist-to-hip ratio (mean ± SD)	0.88 ± 0.05	0.87 ± 0.06	0.89 ± 0.08	> 0.05	
Aspirin reaction units visit 1 (mean ± SD)	489.0 ± 73.0^a^	488.2 ± 83.0^a^	638.3 ± 15.9^b^	= 0.001	
Aspirin reaction units visit 2 (mean ± SD)	487.62 ± 77.4^a^	460.10 ± 82.3^a^ †	639.2 ± 16.3^b^	= 0.001	

### Scheme of the study

**Fig. 1:**
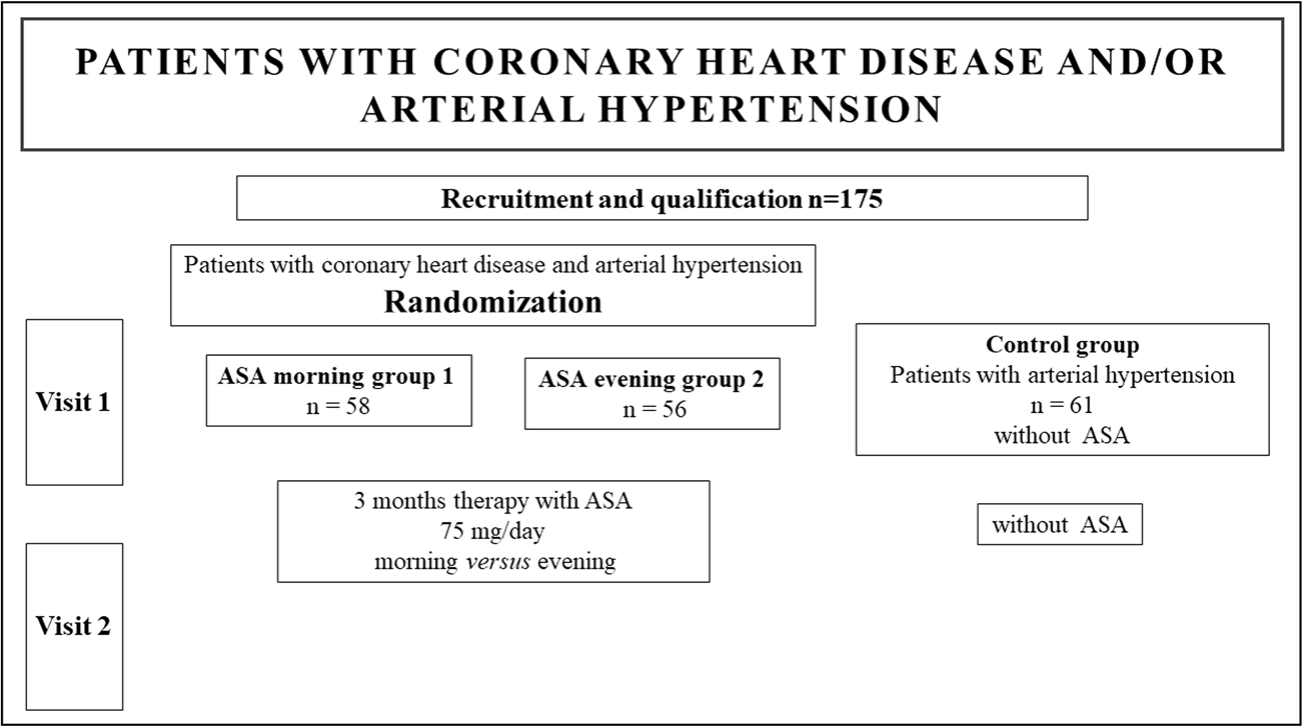
Scheme of the study. Following measurements were performed in each group: laboratory tests, abdominal ultrasound examination, abdominal CT scan, Doppler ultrasound of renal arteries, clinical BP (3x/24 h), ABPM, ECG, echocardiography, weight, and body mass index assessment

Patients enrolled in the study had two visits in accordance with the prescribed treatment schedule (Fig. 1). On the 1 qualifying visit, the patients had been admitted to the ward where the laboratory tests, clinical blood pressure and ambulatory blood pressure measurements, imaging examinations and assessment of platelet aggregation using the VerifyNow Aspirin test took place. The randomisation regarding the inclusion of ASA morning or evening was carried out. After 3 months of ASA therapy, Visit 2 was held, during which the examinations from the initial visit were repeated.

### Blood pressure measurements

In all the patients, during each visit, clinical blood pressure measurements were performed three times at rest, in a supine position, in standard conditions, using an upper-arm blood pressure monitor (Omron 705IT). Ambulatory blood pressure measurements were carried out using an A&D 24-h ambulatory peripheral blood pressure monitor. The frequency of measurements was every 15 min between 7:00 and 22:00, and every 30 min between 22:00 and 7:00. Subsequently, mean arterial pressure (MAP) was calculated from the formula MAP = DBP + 1/3 (SBP – DBP) [mm Hg].

Mean diurnal and nocturnal values of SBP and DBP were analysed. The percentage drop in blood pressure was calculated using the following equations:

$$ {\displaystyle \begin{array}{c}\%\mathrm{drop}\ \mathrm{in}\ \mathrm{SBP}=\left[\left(\mathrm{SBP}\ \mathrm{day}\hbox{--} \mathrm{SBP}\ \mathrm{night}\right)/\mathrm{SBP}\ \mathrm{day}\right]\times 100\%\\ {}\%\mathrm{drop}\ \mathrm{in}\ \mathrm{DBP}=\left[\left(\mathrm{DBP}\ \mathrm{day}\hbox{--} \mathrm{DBP}\ \mathrm{night}\right)/\mathrm{DPB}\ \mathrm{day}\right]\times 100\%\end{array}} $$

The night blood pressure fall was calculated as follows:

$$ \mathrm{NBPF}=\left[\left(\mathrm{MAP}\ \mathrm{day}\hbox{---} \mathrm{MAP}\ \mathrm{night}\right)/\mathrm{MAP}\ \mathrm{day}\right)\Big]\times 100\% $$

Patients with normal NBPF (10–20%) were referred to as “dippers.” Patients with NBPF < 10% were classified as “non-dippers,” and patients with NBPF exceeding 20% were classified as “extreme dippers” [30, 31].

### Verify Now Aspirin test

The Verify Now Aspirin Test method was described in our previous manuscript published in the Cardiology Journal [32].

### Statistical analysis

Statistical analyses were performed with Statistica, version 13.0. (StatSoft, USA). Since the tested data had not met the assumption of Gaussian distribution (evaluated with Shapiro–Wilk method), non-parametric methods were applied. The Kruskal-Wallis ANOVA test was used for evaluation of the differences in parameters between the three groups. The multiple comparison post-hoc Dunn’s test was performed in case *p* value for Kruskal-Wallis ANOVA was below 0.05—in such a case a use of different superscripts in tables was applied to denote statistically significant differences between compared groups. Changes in parameters in the individual groups during the first and second visit were analysed using the Wilcoxon signed-rank test—the parametric equivalent of the *t* test for related variables. Differences between two independent groups were tested with Mann-Whitney U test. Frequencies, expressed on a nominal scale, were analysed based on the Pearson chi-squared test. To evaluate differences in number of dippers, extreme dippers and non-dippers in each group at Visit 1 and Visit, the Fisher’s exact test was performed. A *p* value < 0.05 was considered significant.

## Results

**Fig. 2:**
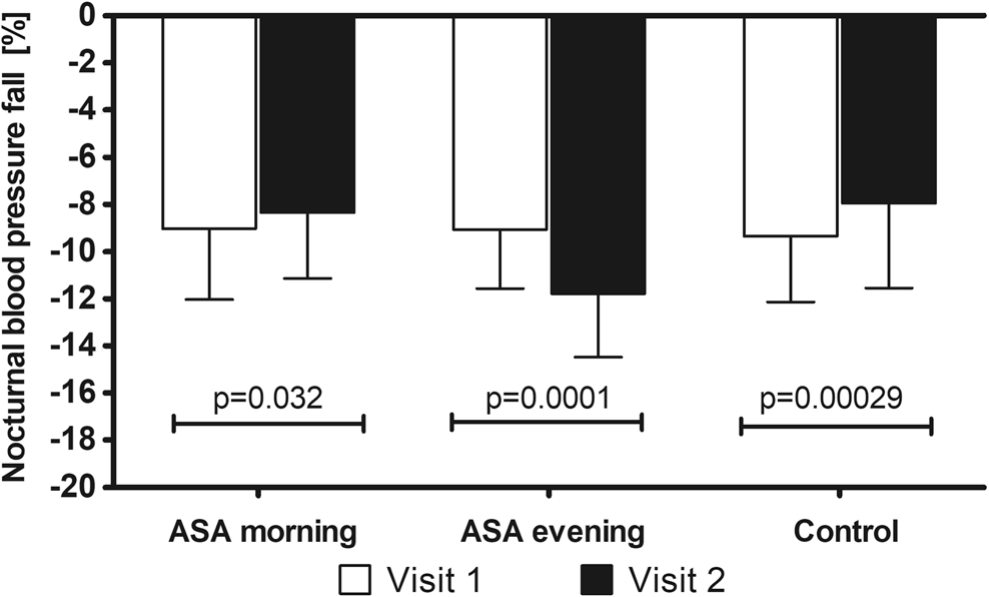
Changes of nocturnal blood pressure fall between Visit 1 and Visit 2 in the studied groups (*p* value refers to Wilxocon-signed rank test)

**Fig. 3:**
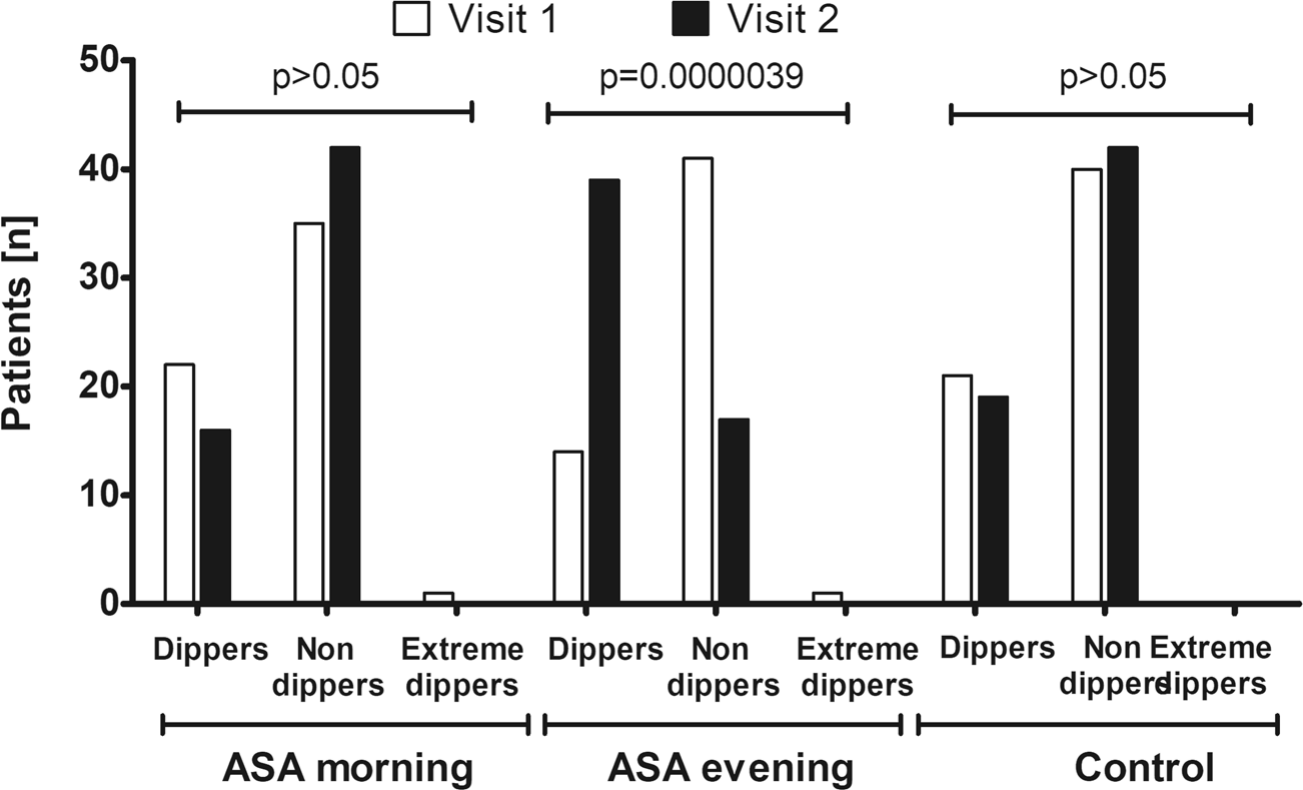
The number of non-dipping, dipping, and extremely dipping patients in the studied groups during the Visit 1 and Visit 2 (p value refers to Fisher’s exact test)

The basic demographical and clinical characteristics of studied groups of patients are summarized in Table 1 and Table 2. Selected parameters such as body mass index (BMI), weight, waist, and hips did not differ significantly between the studied groups and did not change considerably between Visits 1 and 2 (*p* > 0.05, the Wilcoxon signed-rank test). More importantly, the studied groups did not differ significantly in the amount of administered drugs (*p* > 0.05, the Pearson chi-squared test), and there was no change of medications during the study. The results of daytime, night time, and average 24-h BP parameters in the ABPM at the first and second visits are presented in Table 3. As shown, only a group receiving ASA in the evening for 3 months revealed a significant decrease in the blood pressure at night (SBPn by 5.5 mmHg, DBPn by 4.6 mmHg and MAPn by 4.9 mmHg) and in the 24-h BP measurements (SBP24 by 5.5 mmHg, DBP24 by 3.56 mmHg and MAP24 by 4.2 mmHg). Although the group receiving ASA in the morning revealed a decrease in the clinic BP (by 2.5/1.7 mmHg), the statistically higher drop was displayed by patients using ASA in the evening (by 5.4/3.8 mmHg) (Table 3). As shown in Table 3 and Fig. 2, only the group receiving ASA in the evening experienced a significant increase in the NBPF between Visit 1 and Visit 2 (by mean of 3.5%), while in the control group and the group with morning ASA administration, the opposite trend was noted. A mean NBPF at Visit 2 was 8.3 and 7.9% in the ASA morning and control group, respectively, and 11.8% in the ASA evening group. A beneficial change observed in the latter group was relevant enough to significantly increase the number of dippers in this group—nearly threefold, from 14 patients at Visit 1 to 39 patients at Visit 2. In the case of the group with morning ASA administration, a decrease in patients identified as dippers was observed—from 22 at Visit 1 to only 16 at Visit 2 (Fig. 3). The statistically significant difference in BMI between non-dippers and dippers was revealed in the ASA evening group with a mean ± SD of 31.4 ± 4.4 and 28.6 ± 44.1 (Mann-Whitney U test, *p* = 0.04). The laboratory results for each group at Visit 1 and Visit 2 are presented in Table S1. None of the considered parameters was altered in all groups except uric acid, which concentration at Visit 2 was increased in the group receiving ASA in the evening when compared with that at Visit 1 (Table S1).

**Table 2:** Comorbidities and pharmaceuticals used in the studied group

	ASA morning	ASA evening	Control group
Comorbidity (*n*)			
Coronary heart disease	58	56	0
Arterial hypertension	58	56	61
Diabetes mellitus	15	17	14
Metabolic syndrome	21	20	22
History of myocardial infarction	12	13	0
Previous ischaemic stroke or transient cerebral ischaemia	5	6	0
History of coronary artery bypass surgery	8	9	0
History of coronary angioplasty	38	37	0
Hyperlipidaemia	58	56	61
Atherosclerosis of the lower limbs	7	6	5
Chronic obstructive pulmonary disease	6	7	5
Thyroid disease	4	5	5
Drugs (*n*)			
Acetylsalicylic acid	58	56	0
Anti-hypertensive drugs	58	56	57
β-blockers	58	56	53
Angiotensin-converting enzyme inhibitors	40	34	40
Angiotensin II receptor antagonists	18	22	21
Calcium antagonists	22	25	25
Diuretics/aldosterone antagonists	13	12	16
Concomitant lipid-lowering therapy	58	56	58
Concomitant antidiabetic therapy (metformin)	15	17	14
Proton pump inhibitors	47	45	42

**Table 3:** The results of office blood pressure measurements and parameters of 24-h monitoring (mean ± SD) in the studied groups at Visits 1 and Visit 2. Different superscript letters (a, b) denote significant differences between groups in each row (*p* < 0.05 in multiple comparison post-hoc Dunn test following Kruskal-Wallis ANOVA)

		Kruskal Wallis ANOVA	ASA morning	ASA evening	Control group
SBP d [mm Hg]	Visit 1	> 0.05	139.1 ± 6.5	139.4 ± 7.2	139.2 ± 6.1
	Visit 2	> 0.05	137.9 ± 5.5	136.5 ± 6.7	137.8 ± 5.2
	p (Wilcoxon)		***p = 0.0014***	***p = 0.0001***	***p = 0.0001***
DBP d [mm Hg]	Visit 1	> 0.05	86.5 ± 5.2	86.4 ± 5.4	86.8 ± 5.1
	Visit 2	> 0.05	84.9 ± 5.2	84.3 ± 6.2	85.7 ± 5.5
	p (Wilcoxon)		***p = 0.0048***	***p = 0.0001***	***p < 0.0001***
MAP d [mm Hg]	Visit 1	> 0.05	104.1 ± 4.5	104.1 ± 4.9	104.3 ± 4.2
	Visit 2	> 0.05	102.6 ± 4.0	101.7 ± 5.2	102.4 ± 4.5
	p (Wilcoxon)		***p = 0.0001***	***p = 0.0001***	***p = 0.0001***
SBPn [mm Hg]	Visit 1	> 0.05	125.7 ± 7.3	125.6 ± 7.0	125.2 ± 6.8
	Visit 2	< 0.05	125.2 ± 7.0 ^a^	120.1 ± 6.9 ^b^	125.7 ± 6.6 ^a^
	p (Wilcoxon)		*p* > 0.05	***p = 0.0001***	*p < 0.05*
DBPn [mm Hg]	Visit 1	> 0.05	79.5 ± 5.3	79.1 ± 4.7	79.1 ± 4.9
	Visit 2	< 0.05	78.4 ± 4.6 ^a^	74.5 ± 5.3 ^b^	78.4 ± 5.2 ^a^
	p (Wilcoxon)		*p > 0.05*	***p = 0.0001***	*p > 0.05*
MAPn [mm Hg]	Visit 1	> 0.05	94.6 ± 4.2	94.6 ± 4.0	94.5 ± 4.1
	Visit 2	*< 0.05*	94.0 ± 3.8 ^a^	89.7 ± 4.6 ^b^	94.2 ± 4.0 ^a^
	p (Wilcoxon)		*p > 0.05*	***p = 0.0001***	*p > 0.05*
SBP 24 [mm Hg]	Visit 1	> 0.05	134.6 ± 6.2	134.4 ± 6.8	134.6 ± 6.3
	Visit 2	< 0.05	132.8 ± 5.7 ^a^	128.9 ± 5.8 ^b^	134.2 ± 4.9 ^a^
	p (Wilcoxon)		*p > 0.05*	***p = 0.0001***	*p > 0.05*
DBP 24 [mm Hg]	Visit 1	> 0.05	82.6 ± 4.4	82.3 ± 4.8	83.2 ± 5.1
	Visit 2	< 0.05	82.3 ± 4.1 ^a^	78.7 ± 5.9 ^b^	83.1 ± 5.5 ^a^
	p (Wilcoxon)		*p > 0.05*	***p = 0.0001***	*p > 0.05*
[mm Hg]	Visit 1	> 0.05	100.0 ± 3.9	99.7 ± 4.4	100.3 ± 4.6
	Visit 2	< 0.05	99.2 ± 3.6 ^a^	95.5 ± 5.1 ^b^	100.2 ± 4.3 ^a^
	p (Wilcoxon)		*p > 0.05*	***p = 0.0001***	*p > 0.05*
NBPF [%]	Visit 1	> 0.05	9.0 ± 3.0	9.1 ± 2.5	9.3 ± 2.8
	Visit 2	< 0.05	8.3 ± 2.8 ^a^	11.8 ± 2.7 ^b^	7.9 ± 3.6 ^a^
	p (Wilcoxon)		***p = 0.032***	***p = 0.0001***	***p = 0.0002***
Clinic SBP [mm Hg]	Visit 1	> 0.05	145.5 ± 5.0	145.2 ± 6.7	145.1 ± 5.2
	Visit 2	< 0.05	143.0 ± 4.1 ^a^	139.8 ± 5.6 ^b^	144.2 ± 4.6 ^a^
	p (Wilcoxon)		***p = 0.0001***	***p = 0.0001***	*p > 0.05*
Clinic DBP [mm Hg]	Visit 1	> 0.05	88.8 ± 4.0	88.8 ± 5.3	89.0 ± 5.5
	Visit 2	< 0.05	87.2 ± 4.7 ^a^	85.0 ± 5.7 ^b^	87.9 ± 3.0 ^a^
	p (Wilcoxon)		***p = 0.004***	***p = 0.0001***	*p > 0.05*

SBPd, ambulatory daytime systolic blood pressure; DBPd, ambulatory daytime diastolic blood pressure; MAPd, ambulatory daytime mean blood pressure; SBPn, ambulatory nighttime systolic blood pressure; DBPn, ambulatory nighttime diastolic blood pressure; MAPn, ambulatory nighttime mean blood pressure; SBP24, 24-h systolic blood pressure; DBP24, 24-h diastolic blood pressure; MAP24, 24-h ambulatory mean blood pressure; NBPF, night blood pressure fall; clinic SBP, systolic blood pressure; clinic DBP, diastolic blood pressure

## Discussion

The present study demonstrated the clinic blood pressure drop after 3 months of evening ASA administration in patients with arterial hypertension coronary heart disease who were already taking ASA. More importantly, the same has been confirmed in ABPM. There was a significant reduction in the average 24-h BP (SBP24, DBP24 and MAP24) and night blood pressure (SBPn, DBPn, MAPn) in the ASA evening group compared with the ASA morning group and the control group. Interestingly, during the day hours, there was a statistically significant decrease in systolic, diastolic, and average 24-h BP in both groups: ASA morning and ASA evening. This could be due to the antihypertensive drugs used by patients on their own although the nocturnal BP drop found only in the evening group highlights a favourable chronotherapeutic effect of ASA and advocates its use at bedtime in subjects with arterial hypertension and coronary heart disease.

The anti-hypertensive mechanism of ASA is not fully understood. During the night, there is a physiological drop in blood pressure, which causes a decrease in perfusion through the kidneys, and this is a signal for the activation of the renin-angiotensin-aldosterone (RAA) system. The beneficial effect of ASA administration in the evening can be explained by the effect of inhibiting the increase of RAA system activity, as well as the beneficial effects on the production of nitric oxide [33]. Snoep et al. have shown that taking ASA at bedtime compared with morning administration reduced the mean plasma renin activity without affecting aldosterone levels. The excretion of cortisol, dopamine, and norepinephrine in diurnal urine collection was lower in the group of patients treated with ASA given in the evening. The activity of RAA system, cortisol levels, and catecholamines follows a circadian rhythm, peaking in the early morning hours. This allows the assumption that the administration of ASA in the morning would be too late. Thus, taking ASA at bedtime can be considered as the right moment to harness the additional anti-hypertensive effects of this drug [34].

The results published by Hermida et al., which refer to a group of 328 subjects with mild, non-treated hypertension, are concordant with the results obtained in the present study. In the referenced study, after 3 months of ASA administration in the morning and in the evening, in the ASA evening group a significant drop in blood pressure was obtained in clinical and ABPM measurements. ASA belongs to the group of non-steroidal anti-inflammatory drugs, which by inhibiting cyclooxygenase type 1 and type 2 causes an increase in blood pressure [3, 18, 35]. Therefore, the results obtained by Hermida et al. were considered surprising and unexpected. However, the same researcher team obtained similar results in three consecutive studies in pregnant women and patients with prehypertension [36–38]. In turn, the present study adds that ASA administration at bedtime, contrary to morning ingestion, results in better effect on blood pressure in patients suffering from arterial hypertension and coronary heart disease, who are not naïve to ASA.

Chronotherapy with ASA is poorly understood and contradictory reports have been published so far. In contrast to the results of the present study, Magen et al. described the anti-hypertensive effect of ASA after morning drug administration. They have shown that the addition of low-dose ASA to anti-hypertensive drugs in patients with hypertension and hypercholesterolemia may reduce both SBP and DBP as well as improve endothelial function, measured by the ability of the brachial artery to dilate [39]. The probable mechanism by which ASA lowers BP is its effect on reducing vasoconstriction through inhibition of TXA2 production, as well as increasing production of nitric oxide causing vasodilation. However, in our previous study, we have not found any reduction in blood pressure following a 4-week treatment with ASA compared with standardized tomato extract (213 mg/day). Both drugs were administered in the morning, and the treatment group included patients of high and very high cardiovascular risk [40]. Similar results were published by Avazini et al. who did not observe a decrease in SBP and DBP in patients treated for hypertension with a low-dose ASA administered in the morning [19]. In a randomized study, a group of 290 patients showed no decrease in blood pressure despite the use of ASA in the evening, while a reduction in platelet reactivity was observed in the Verify Now Aspirin test [41]. The above data suggest that the effect of ASA on blood pressure may be dependent on the time of dosing and independent of antiplatelet activity.

Through the use of ABPM, this study draws attention to the effect of ASA not only on a single blood pressure measurement but also on the diurnal blood pressure profile. Compared with the ASA morning group, in the ASA evening group there was a significant increase in nocturnal blood pressure drop. Paradoxically, in the ASA morning group, there was a slight decrease in the NBPF value, which meant that the patients still had a non-dippers profile. This observation may be of clinical significance because for patients in whom the pressure drop at the night does not exceed 10–20% (non-dippers); there exists a greater risk of developing subclinical organ damage [14, 42]. The relationship between lack of blood pressure drop at night and risk of cardiovascular events was confirmed in many studies [12, 16, 43]. In their studies, Verdecchia et al. and Syst-Eur showed that non-dippers hypertensive patients experienced more frequent cardiovascular events, compared with the dippers group [44–46]. Hermida et al. showed that patients who received ASA at bedtime demonstrated a more significant BP reduction at night in the non-dippers group. Together with the current results, this can be considered a confirmation of the time-dependent dosage of ASA on blood pressure, mainly in non-dippers [47]. The data obtained in the current study suggest a significant effect and role of bedtime ASA administration in the group of patients with coronary heart disease and arterial hypertension. The demonstrated hypotensive effect of ASA when used in the evening allows ASA to be included in the group of chronotherapeutics, i.e., the drugs with a time-dependent effect. Interestingly, in our previous work, we showed a significant reduction in platelet aggregation by 28 ARUs in the ASA evening group, which was determined using the Verify Now analyser. At baseline visit, the results of inhibition of aggregation in ARUs did not differ between the groups. This confirms the effect of a low-dose ASA taken at bedtime on the reduction of COX-1-dependent platelet activity. Since the anti-aggregation and anti-hypertensive effect occurred only in the patients taken ASA at bedtime, this may indicate that the mechanism of BP drop and platelet function is common and probably can be associated with COX-1 inhibition [32]. Interestingly, the non-dipping patients in the group receiving ASA at bedtime displayed significantly higher BMI as compared with the individuals revealing dipping pattern. The present study has employed enteric coated form of ASA (which is the most prevalent form of ASA in Poland) and previous investigations has shown that patients’ weight can modify the response to this form—particularly individuals with higher Weight may be less susceptible to its effects [48, 49].

There are number of hypothetical explanations for the observed night drop in BP following the bedtime administration of ASA. Firstly, it may be possible that the activity of COX in endothelium and other tissues is higher during the night, thus explaining the more profound effect of ASA on its inhibition. Secondly, if BP at night is associated with higher platelets activation, the anti-platelet action of ASA may explain the night time reduction in BP. Finally, there may be a difference in ASA metabolism between night and day, with higher ASA inactivation to salicylate during the day. Indeed, studies addressing the circadian rhythms on the kinetics of drugs indicate that their night time metabolism is usually significantly lower [50] Therefore, the night time may represent a window in which ASA effects on COX and platelets may be more profound, eventually inducing a drop in BP. However, all of these hypothesis require further studies to understand the exact mechanisms behind the night time effect of ASA on BP. It would be of high interest to conduct a study with and similar model in which clopidogrel is used instead of ASA. Clopidogrel is a more potent irreversible anti-platelet agent which binds to P2Y12 ADP receptor although it does not inhibit COX [51]. This would allow to test to which extent COX or platelets activity during the night time are involved in changes of BP at night in human. Further valuable information could be derived by including, additionally to platelet aggregometry, an investigation of serum thromboxane B2 levels which can provide a direct measure of the pharmacological effect of ASA and may be even more predictive when performed in tandem with a global measure of platelet function [49, 52].

## Conclusion

The present study demonstrated that compared with the use of ASA in the morning, its administration in the evening may lead to favourable drop in the ABPM and an improvement of the diurnal profile in the high-risk group of cardiovascular patients suffering from arterial hypertension and coronary heart disease who are not naïve to ASA.

## Electronic supplementary material

ESM 1 (DOCX 24 kb)
