# Supplementary material for: The impact of acetylsalicylic acid dosed at bedtime on circadian rhythms of blood pressure in the high-risk group of cardiovascular patients—a randomized, controlled trial
Source: Eur J Clin Pharmacol. 2020 Sep 21;77(1):35–43. doi: 10.1007/s00228-020-02997-8 (PMC7782434; doi:10.1007/s00228-020-02997-8)
Supplement: Supplementary file 1 — (DOCX 24 kb) [file 228_2020_2997_MOESM1_ESM.docx]

Table S1. The results of laboratory tests in the studied groups at Visit 1 and Visit 2 (mean±SD). Different superscripts in a row denote significant differences between groups (p < 0.05; post-hoc Dunn test following Kruskal-Wallis ANOVA)

|  |  | ASA morning | ASA evening | Control group |
| --- | --- | --- | --- | --- |
| Hemoglobin [mmol/L] | Visit 1 | 8.8±0.74 ^a^ | 8.7±0.58 ^a^ | 8.9±0.73 ^a^ |
|  | Visit 2 | 8.8±0.7 ^a^ | 8.6±0.7 ^a^ | 8.9±0.7 ^a^ |
|  | p(Wilcoxon) | *p>0.05* | *p>0.05* | *p>0.05* |
| Platelet [10^-9^/L] | Visit 1 | 225.7±54.3 ^a^ | 227.6±56.9 ^a^ | 224.5±61.1 ^a^ |
|  | Visit 2 | 226.9±36.8 ^a^ | 197.9±41.5 ^b^ | 226.0±60.2 ^a^ |
|  | p(Wilcoxon) | *p>0.05* | *p<0.05* | *p>0.05* |
| Sodium [mmol/L] | Visit 1 | 141.2±2.5 ^a^ | 141.1±2.2 ^a^ | 141.7±2.1 ^a^ |
|  | Visit 2 | 141.0±2.1 ^a^ | 140.9±2.2 ^a^ | 141.9±2.3 ^a^ |
|  | p(Wilcoxon) | *p>0.05* | *p>0.05* | *p>0.05* |
| Potassium [mmol/L] | Visit 1 | 4.3±0.45 ^a^ | 4.3±0.4 ^a^ | 4.2±0.4 ^a^ |
|  | Visit 2 | 4.3±0.3 ^a^ | 4.3±0.4 ^a^ | 4.2±0.3 ^a^ |
|  | p(Wilcoxon) | *p>0.05* | *p>0.05* | *p>0.05* |
| Total cholesterol [mmol/L] | Visit 1 | 4.8±1.1 ^a^ | 4.7±1.0 ^a^ | 4.7±0.9 ^a^ |
|  | Visit 2 | 4.7±0.9 ^a^ | 4.8±1.1 ^a^ | 4.4±0.9 ^a^ |
|  | p(Wilcoxon) | *p>0.05* | *p>0.05* | *p>0.05* |
| Low-density lipoprotein cholesterol [mmol/L] | Visit 1 | 1.3±0.4 ^a^ | 1.4±0.5 ^a^ | 1.4±0.5 ^a^ |
|  | Visit 2 | 1.5±0.5 ^a^ | 1.5±0.6 ^a^ | 1.7±0.6 ^a^ |
|  | p(Wilcoxon) | *p>0.05* | *p>0.05* | *p>0.05* |
| High-density lipoprotein cholesterol [mmol/L] | Visit 1 | 2.6±1.0 ^a^ | 2.5±0.9 ^a^ | 2.5±0.7 ^a^ |
|  | Visit 2 | 2.5±0.8 ^a^ | 2.4±0.8 ^a^ | 2.5±0.7 ^a^ |
|  | p(Wilcoxon) | *p>0.05* | *p>0.05* | *p>0.05* |
| Triglycerides [mmol/L] | Visit 1 | 1.6±0.5 ^a^ | 1.6±0.4 ^a^ | 1.5±0.8 ^a^ |
|  | Visit 2 | 1.6±0.4 ^a^ | 1.5±0.5 ^a^ | 1.7±0.8 ^a^ |
|  | p(Wilcoxon) | *p>0.05* | *p>0.05* | *p>0.05* |
| Glomerular filtration rate [mL/min/1.73 m^2^] | Visit 1 | 80.4±9.7 ^a^ | 79.3±12.6 ^a^ | 78.8±11.7 ^a^ |
|  | Visit 2 | 80.6±9.0 ^a^ | 82.9±8.9 ^a^ | 79.7±9.9 ^a^ |
|  | p(Wilcoxon) | *p>0.05* | *p>0.05* | *p>0.05* |
| Serum creatinine [µmol/L] | Visit 1 | 82.2±5.1 ^a^ | 82.2±5.1 ^a^ | 81.6±5.2 ^a^ |
|  | Visit 2 | 79.4±15.4 ^a^ | 82.9±12.0 ^a^ | 82.3±10.9 ^a^ |
|  | p(Wilcoxon) | *p>0.05* | *p>0.05* | *p>0.05* |
| Glucose [mmol/L] | Visit 1 | 5.2±0.9 ^a^ | 5.2±0.7 ^a^ | 5.2±0.6 ^a^ |
|  | Visit 2 | 5.1±0.7 ^a^ | 5.3±0.7 ^a^ | 5.1±0.5 ^a^ |
|  | p(Wilcoxon) | *p>0.05* | *p>0.05* | *p>0.05* |
| Urid acid [µmol/L] | Visit 1 | 331.9±101 ^a^ | 334.3±99 ^a^ | 330.7±97 ^a^ |
|  | Visit 2 | 332.7±105 ^a^ | 379.1±71 ^b^ | 328.9±99 ^a^ |
|  | p(Wilcoxon) | *p>0.05* | *p=0.0057* | *p>0.05* |
| C-reactive protein [mg/L] | Visit 1 | 2.0±1.8 ^a^ | 1.9±0.9 ^a^ | 1.9±0.8 ^a^ |
|  | Visit 2 | 2.1±1.6 ^a^ | 2.1±1.0 ^a^ | 1.8±0.8 ^a^ |
|  | p(Wilcoxon) | *p>0.05* | *p>0.05* | *p>0.05* |
